# Nonrigid registration with corresponding points constraint for automatic segmentation of cardiac DSCT images

**DOI:** 10.1186/s12938-017-0323-1

**Published:** 2017-03-28

**Authors:** Xuesong Lu, Rongqian Yang, Qinlan Xie, Shanxing Ou, Yunfei Zha, Defeng Wang

**Affiliations:** 1 College of Biomedical Engineering, South-Central University for Nationalities, Wuhan, 430074 People’s Republic of China; 2 School of Materials Science and Engineering, South China University of Technology, Guangzhou, 510006 People’s Republic of China; 3 Radiology Department, Guangzhou General Hospital of Guangzhou Military Area Command, Guangzhou, 510010 People’s Republic of China; 4 Department of Radiology, Remin Hospital of Wuhan University, Wuhan, 430060 People’s Republic of China; 5 Research Center for Medical Image Computing, Department of Imaging and Interventional Radiology, The Chinese University of Hong Kong, Shatin, New Territories, Hong Kong, China; 6 Shenzhen Research Institute, The Chinese University of Hong Kong, Shenzhen, China

**Keywords:** Dual-source computed tomography (DSCT), Nonrigid registration, Mutual information, Corresponding points, *n*-dimensional scale invariant feature transform (*n*-SIFT), Automatic heart segmentation

## Abstract

**Background:**

Dual-source computed tomography (DSCT) is a very effective way for diagnosis and treatment of heart disease. The quantitative information of spatiotemporal DSCT images can be important for the evaluation of cardiac function. To avoid the shortcoming of manual delineation, it is imperative to develop an automatic segmentation technique for 4D cardiac images.

**Methods:**

In this paper, we implement the heart segmentation-propagation framework based on nonrigid registration. The corresponding points of anatomical substructures are extracted by using the extension of *n*-dimensional scale invariant feature transform method. They are considered as a constraint term of nonrigid registration using the free-form deformation, in order to restrain the large variations and boundary ambiguity between subjects.

**Results:**

We validate our method on 15 patients at ten time phases. Atlases are constructed by the training dataset from ten patients. On the remaining data the median overlap is shown to improve significantly compared to original mutual information, in particular from 0.4703 to 0.5015 ($p = 5.0 \times 10^{ - 4}$) for left ventricle myocardium and from 0.6307 to 0.6519 ($p = 6.0 \times 10^{ - 4}$) for right atrium.

**Conclusions:**

The proposed method outperforms standard mutual information of intensity only. The segmentation errors had been significantly reduced at the left ventricle myocardium and the right atrium. The mean surface distance of using our framework is around 1.73 mm for the whole heart.

## Background

In recent years, the morbidity of cardiovascular diseases (CVDs) is rapidly increasing in China. An estimated 3.0 million Chinese died from CVDs in 2011, accounting for 41% of all deaths [1]. An early diagnosis and treatment for this illness is of great use to reduce the death toll. Although the doctors diagnose it by electrocardiogram or imaging of patient, the shortcoming of these means is absence of quantitative information. Recent advances in evaluation of cardiac function based on medical images have shown tremendous potential towards achieving quantitative diagnosis [2, 3]. Among the various imaging modalities, cardiac magnetic resonance imaging (MRI) is a mainstream technology because of non-ionizing radiation [4].

However, computed tomography (CT) has been widely used in the form of not only 3D images describing the cardiac anatomy but also 3D + time image sequences including anatomical and functional information [5]. Some advanced techniques such as multislice CT (MSCT) [6] and dual-source CT (DSCT) [7] have demonstrated high specificity and distinguishability in cardiac structure. To evaluate the cardiac function, doctors commonly extract the cardiac chambers, large vessels or coronary arteries from a patient scan. Classical manual delineation is no longer suitable for 3D and 3D + time images due to the quantity of data. The other reason is observer variations of manual annotation might affect the reliability and repeatability of quantitative evaluation. It is highly desirable to develop an automatic segmentation for clinical problem.

At present, automatic segmentation methods of the heart fall into two broad categories. Boundary-based segmentation incorporating prior knowledge is just one of those things. Assen et al. [8] presented a 3-D active shape model for semi-automatic segmentation of cardiac CT and MR volumes. A fuzzy c-means based fuzzy inference system was incorporated into the model. A new method was proposed for the local assessment of boundary detection [9]. It took any boundary detection function and evaluated its performance for a single model landmark in terms of an estimated geometric boundary detection error. The authors demonstrated this method can automatically segment computed tomography and magnetic resonance images. Shang introduced a novel scheme for the segmentation of 4-D MR cardiac images [10]. 3D spatially hierarchical expressions of the statistical shape models for the cardiac chambers were constructed through principal component analysis (PCA) of the manually segmented training set. The limitation of these approaches is that the surfaces from different substructures of the heart are prone to intersect each other in segmentation results. An automatic method was proposed to segment the left ventricles and then identify their borders robustly [11]. The strengths of four techniques: automatic threshold selection, boundary extraction, deformation flow tracking, and convex shape modeling were effectively combined. In a review of segmentation methods of cardiac MR images using the short-axis view, the authors proposed the two main categories: segmentation based on no or weak prior, and segmentation based on strong prior [12].

Another popular method, called registration-based segmentation, is to propagate the segmentation of an atlas image using deformation field after registration. Zhuang et al. [13] proposed a fully automatic whole heart segmentation framework. The locally affine registration method and the free-form deformations with adaptive control point status were applied to registration procedure. Peyrat et al. [14] presented a framework for the nonlinear spatiotemporal registration of 4D time-series of images based on the Diffeomorphic Demons algorithm. The authors declared that registration should be consistent over time as opposed to 3D registration which solely aims at mapping homologous points at a given time-point. A novel multi-atlas segmentation incorporating the intensity, gradient and contextual information was suggested for cardiac MR images [15]. Experimental results show that the accuracy of multi-atlas segmentation can be significantly improved by using the augmented feature vector. Berendsen et al. [16] proposed a new registration with application to organ segmentation in cervical MR. A statistical model, trained on the shapes of a set of segmentations, was integrated as a penalty term in a free-form registration framework. Compared with registration without the use of statistical knowledge, the segmentations were significantly improved.

In this paper, we propose a nonrigid registration algorithm with corresponding points constraint. The feature point pairs are extracted from the fixed and moving image by the extension of *n*-dimensional scale invariant feature transform (*n*-SIFT) method. Automatic segmentation of 4D cardiac CT images is implemented by the use of propagation framework based on registration. Our method addresses in the large variations and boundary ambiguity of the heart structures for the registration between subjects. We evaluate the method on fifteen patients at ten time phases, among which the training dataset is from ten patients for atlas construction. The remaining data is used to test the performance of our approach with comparison to original mutual information.

## Methods

**Fig. 1:**
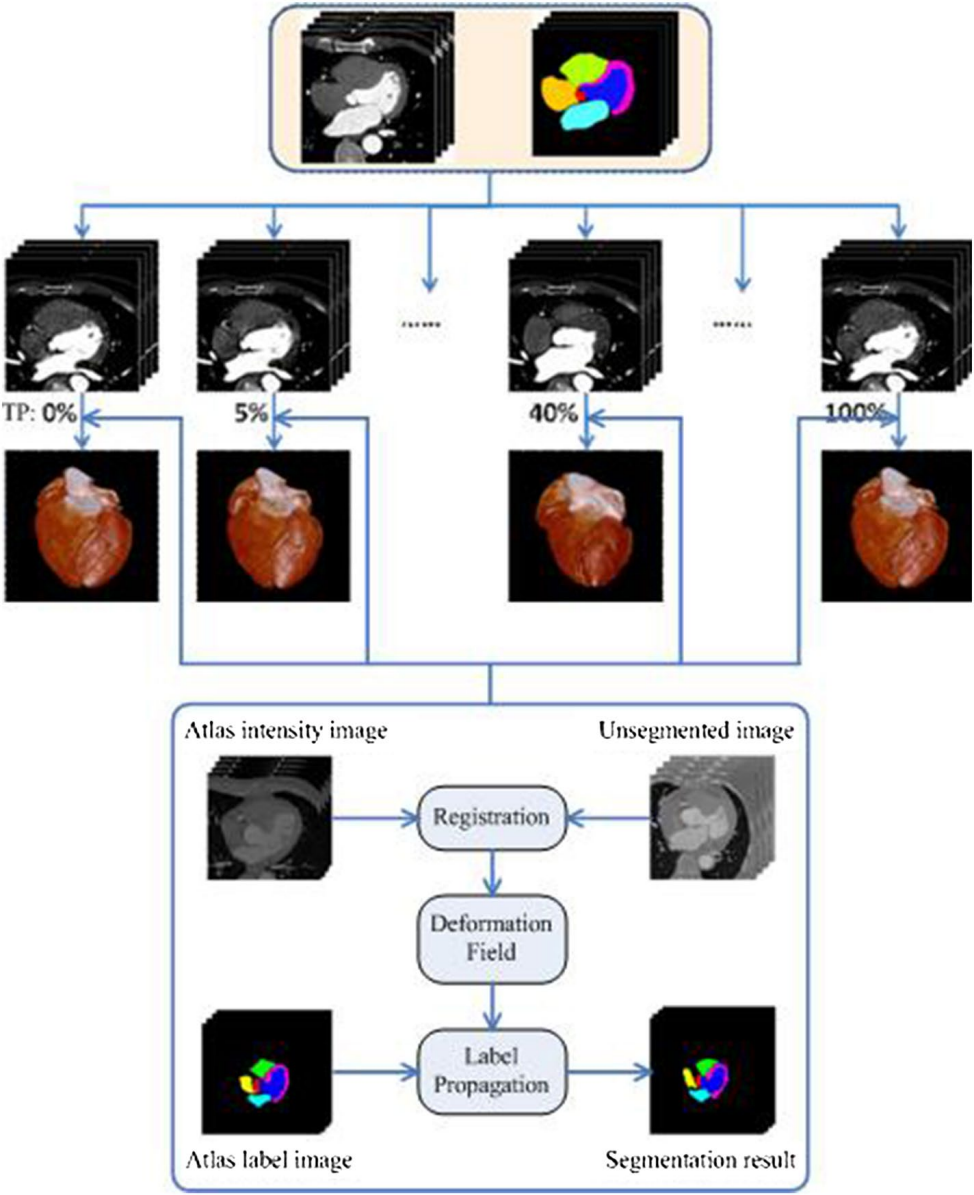
The segmentation-propagation framework of 4D spatiotemporal cardiac CT images based on registration

In this section, we describe the 4D spatiotemporal segmentation-propagation framework of cardiac CT images using registration technique. As shown in Fig. 1, it includes three basic steps. Firstly, atlas intensity images are constructed on the training dataset for each time phase. Atlas label images (different colors represent different substructures) are later produced by manual segmentation. Secondly, we register the images needing segmentation to atlas intensity images with the nonrigid transformation. Finally, the segmentation results are propagated from atlas label images by the deformation field of registration.

### Atlas construction

**Fig. 2:**
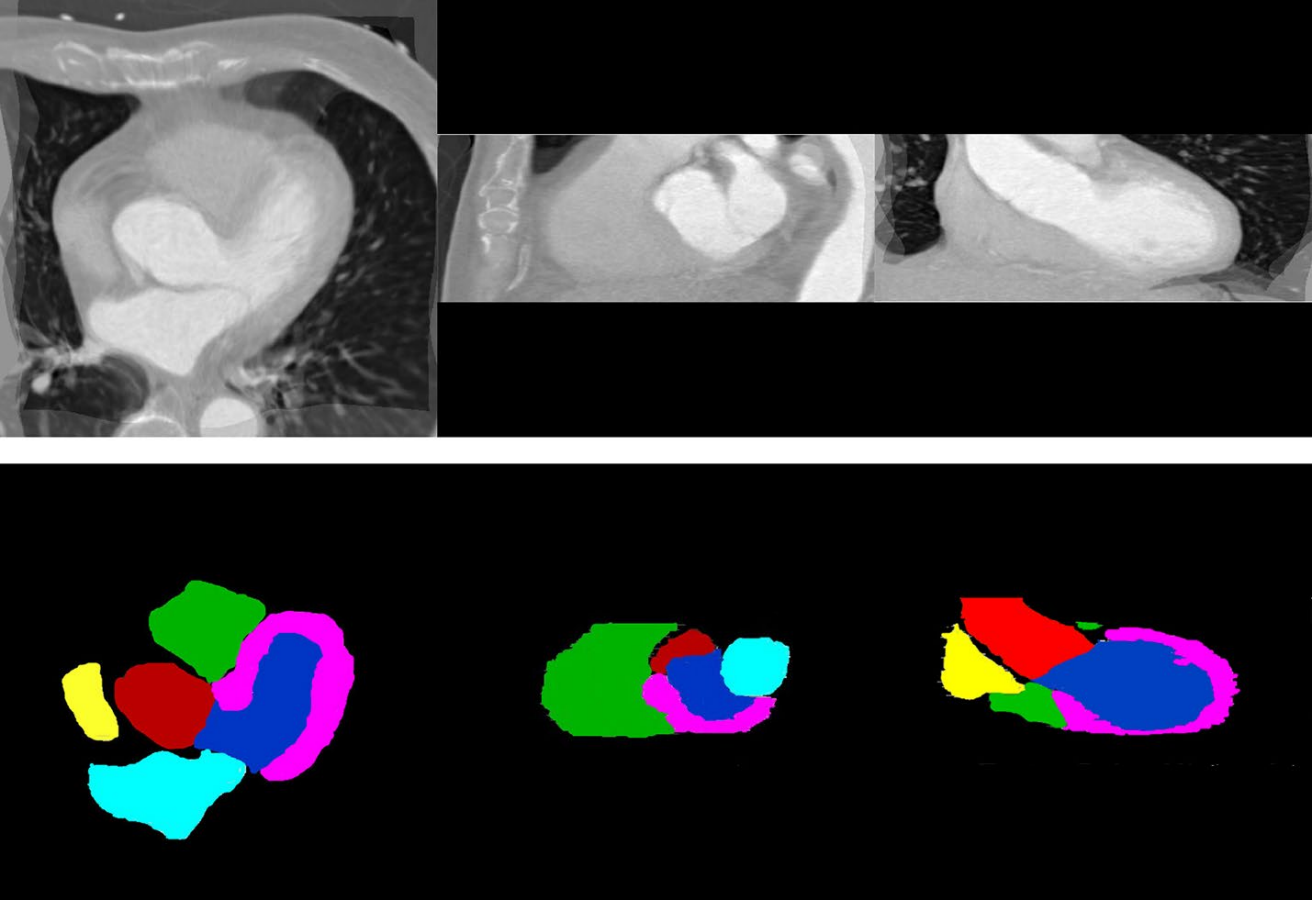
An atlas example that is shown in axial, sagittal, and coronal planes. *Upper* an atlas intensity (CT) image using the mean shape of ten patient data. *Lower* atlas label image including aorta (*crimson*), left atrium (*cyan*), left ventricle (*blue*), left ventricle myocardium (*magenta*), right atrium (*yellow*), and right ventricle (*green*)

Atlas often plays an important role in computational physiology of the heart [17]. It can provide the distinct division and localization of substructures, regarded as a standard reference frame. Atlas construction from a population of images is a difficult and complicated topic. We use a simple atlas similar to Ref. [13] because the main goal of this work is to validate the performance of the proposed framework. In practice, a reference space is selected from a number of CT images at the first. The other CT images are then registered to the reference space. At last, we compute a mean intensity image (called as atlas intensity image) from these transformed CT images based on registration results. The atlas label image can provide corresponding segmentation information of each anatomical substructure of the reference space. Figure 2 shows an atlas case at the first time phase.

In order to produce a high quality atlas, some researchers involved the statistical shape model into registration procedure [6, 18, 19]. A statistical atlas can provide some advantages in the postprocessing and analysis of largely variable datasets. The encoding of population variation indicates that their spatial relationships are known. Here we achieved the registration incorporating shape information of multiple-object for atlas construction so as to avoid the complexity of statistical shape model. The important steps are described as follows:

- A combined transformation *T* = *T*_*global*_ + *T*_*local*_ is employed. The global transformation is an affine model, and the local transformation is a free-form deformation (FFD) model based on B-splines [20].
- α-Mutual information (α-MI) [21] is combined with Kappa Statistic [22] of six substructures, which is regarded as the similarity measure.
- An iterative stochastic gradient descent optimization strategy [23] is used to obtain the optimal deformation field.

### Registration between atlas and unsegmented image

The goal of image registration in this subsection is to relate any point in the atlas intensity image to the unsegmented image. In other words, this purpose is to find the optimal transformation *T*: $(x, y, z, t) \to (x^{\prime } , y^{\prime } , z^{\prime } , t^{\prime } )$. We use coarse-to-fine strategy to perform this nonrigid registration. The affine model is applied to rough alignment of the images. Afterwards, the B-splines FFD model is chosen to estimate local motion parameters. The affine alignment result is considered as the initial parameters of nonrigid registration using the B-splines FFD model.

**Fig. 3:**
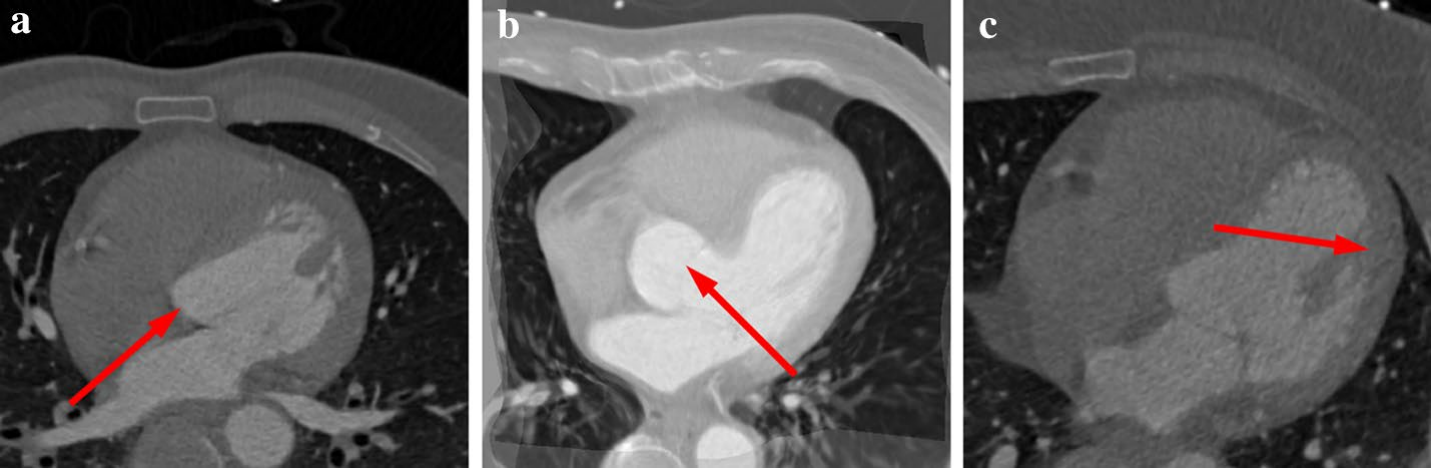
Some limitations for intersubject registration of cardiac substructures. **a**, **b** Demonstrate large changes in left ventricle. **c** Displays boundary ambiguity over left ventricle

The two voxel-based similarity measures, mutual information (MI) and correlation coefficient (CC), have been shown to match images accurately and robustly [24]. MI expresses the amount of information that one image contains about another image. Let *f* be the image intensity of the fixed image, *m* be the image intensity of the moving image, and μ be the transformation parameter. It can be written as follows:

1 $$ MI\left( {\mu ;f,m} \right) = \mathop \sum \nolimits \mathop \sum \nolimits p(f,m;\mu )\log_{2} \left( {\frac{p(f,m;\mu )}{{p_{F} \left( f \right)p_{M} (m;\mu )}}} \right) $$

where *p* is the discrete joint probability, *p*_*F*_ and *p*_*M*_ are the marginal discrete probabilities, computed by summing *p* over the fixed image and the moving image respectively. However, it may not be sufficient for the substructures of cardiac CT images only depending on intensity information. The main reason lies in large variations and boundary ambiguity of some substructures for intersubject registration (see Fig. 3). To overcome these limitations, some researchers imposed statistical shape constraints on nonrigid registration [16, 25]. But it is difficult to implement them in practice due to pre-segmentation prerequisite and high complexity.

To avoid these obstacles, we propose a new registration integrating mutual information and corresponding points. Assume $x_{{F_{i} }}$ and $x_{{M_{i} }}$ are the two point sets with known correspondence, extracted from the fixed and moving image respectively. The regularization term is the Euclidean distance of $x_{{F_{i} }}$ and $x_{{M_{i} }}$. It is defined as:

2 $$ C_{p} = \frac{1}{P}\mathop \sum \nolimits \left\| {x_{{M_{i} }} - T_{\mu } (x_{{F_{i} }} ) } \right\| $$

where *P* is the number of corresponding points, and *T*_*μ*_ is the spatial transformation. So the energy function of registration can be defined as:

3 $$ E = MI + \upomega C_{p} $$

where ω is the weight parameter.

To find the optimal parameters of the B-splines FFD model, we minimize Eq. (3) by the adaptive stochastic gradient descent method. Therefore, the derivative of the energy function *E* with respect to the deformation parameters is required. Its analytical expression can be written as:

4 $$ \frac{\partial }{\partial \mu }E = \frac{\partial MI}{\partial \mu } - \frac{\upomega }{P} \cdot \mathop \sum \nolimits \frac{{(x_{{M_{i} }} - T_{\mu } (x_{{F_{i} }} ))}}{{\left\| {x_{{M_{i} }} - T_{\mu } (x_{{F_{i} }} )} \right\|}} \cdot \frac{\partial T}{\partial \mu }(x_{{F_{i} }} ) $$

### Corresponding points based on *n*-SIFT features

In order to achieve high accuracy of intersubject registration, we extract the feature point pairs from the fixed and moving image by the extension of *n*-SIFT [26] method. In the first step, multi-scale Harris corner and extrema detector in the DoG (Difference of Gaussian) space are used to locate the distinctive points in the unregistered images.

#### Multi-scale Harris corner detector

In general, the Harris interest point detector is not invariant to scale changes. We adopt a new version with automatic scale selection to obtain a scale invariant detector [27]. Let *σ*_*I*_ be the integration scale, *σ*_*D*_ be the differentiation scale, and *L*_*a*_ be the derivative computed in the *a* direction. The scale-adapted autocorrelation matrix of image *I* (*x*, *y*, *z*) is given by:

5 $$ H\left( {x,y,z;\sigma_{I} ,\sigma_{D} } \right) = \sigma_{D}^{2} g\left( {\sigma_{I} } \right)*\left[ {\begin{array}{*{20}c} {L_{x}^{2} (x,y,z,\sigma_{D} )} & {L_{xy} (x,y,z,\sigma_{D} )} & {L_{xz} (x,y,z,\sigma_{D} )} \\ {L_{yx} (x,y,z,\sigma_{D} )} & {L_{y}^{2} (x,y,z,\sigma_{D} )} & {L_{yz} (x,y,z,\sigma_{D} )} \\ {L_{zx} (x,y,z,\sigma_{D} )} & {L_{zy} (x,y,z,\sigma_{D} )} & {L_{z}^{2} (x,y,z,\sigma_{D} )} \\ \end{array} } \right] $$

where *g*(*σ*_*I*_) is the average operator in the neighborhood of the point by smoothing with a Gaussian window of size *σ*_*I*_. A point would be a candidate point if it satisfies

6 $$ \det \left( H \right) - \alpha \cdot trace^{3} \left( H \right) > T_{1} $$

where *T*_1_ is a threshold value.

For the candidate point over scales, it may become a corner point by automatic selection method. The characteristic scale is related to the structure and not to the resolution at which the structure is represented. We compute this operator responses for a set of scales σ_*n*_ using Laplacian-of-Gaussians:

7 $$ LoG\left( {x,y,z;\sigma_{n} } \right) = \sigma_{n}^{2} \left| {L_{xx} \left( {\sigma_{n} } \right) + L_{yy} (\sigma_{n} )} \right| $$

where $\left| \cdot \right|$ denotes the absolute value. If $LoG\left( {x,y,z;\sigma_{i} } \right) > LoG\left( {x,y,z;\sigma_{i - 1} } \right)$, $LoG\left( {x,y,z;\sigma_{i} } \right) > LoG\left( {x,y,z;\sigma_{i + 1} } \right)$, and *LoG*(*x*, *y*, *z*; *σ*_*i*_) > *T*_2_, the point will be considered as the corner point with scale *σ*_*i*_ (*T*_2_ is a threshold parameter).

#### Local extrema detector in the DoG space

Similar to Ref. [26], a multilevel image pyramid is created by down-sample of the Gaussian smoothed image. Therefore, starting from the first image at each level, a series of Gaussian blurred images are generated. For each neighboring pair of blurred images, a DoG image is generated. Within each pyramid level, a voxel of a DoG image is compared with the neighboring voxels, the corresponding voxel in the scale above and all the neighbors, and the corresponding voxel in the scale below and all the neighbors. Finally, we locate extrema with magnitude greater than a threshold *T*_3_.

**Fig. 4:**
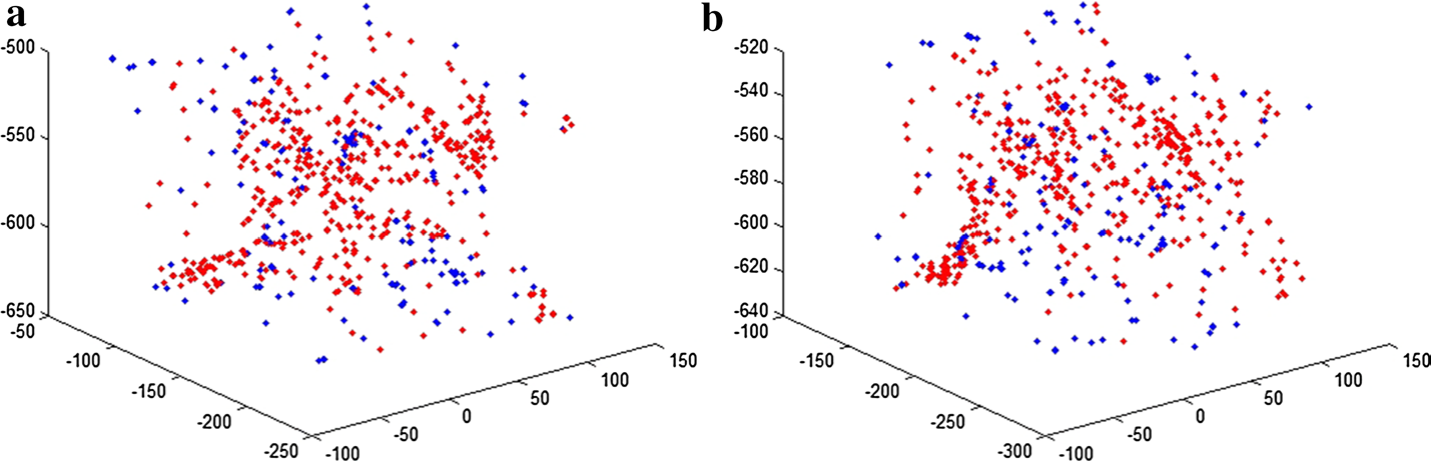
An example of the 3d coordinate distribution of the corresponding points. **a** The feature points from the fixed image. **b** The feature points from the moving image. The *red points* were extracted by local extrema detector in the DoG space, the *blue points* were extracted by multi-scale Harris corner detector

In the second step, the *n*-SIFT feature is generated at each of the extrema position. It divides the local area into 4 × 4 × 4 subregions. A 8 × 8 bin histogram is used to summarize the gradients of the voxels in each subregion. For our experiments, the *n*-SIFT descriptor is a 4096-dimensional feature vector. In the third step, we find the corresponding points from the fixed and moving image by feature matching. The *L*_2_ distances between a feature vector in the fixed image against every feature vector of the moving image are compared to find the best match. A mechanism is employed for removing matches where other features are very close to the best match. Assume that $d(u, v)$ and $d(u, v^{\prime } )$ are the distances from one feature *u* to its nearest feature *v* and next nearest feature *v*′. If the conditional expression

8 $$ \frac{d(u, v)}{{d(u, v^{\prime } )}} < T_{4} $$

is satisfied, *u* and *v* can be considered as a pair of matching feature, where *T*_4_ is a threshold value. Figure 4 displays an example of the 3d coordinate distribution of the corresponding points.

## Experiments

The extraction algorithm of the corresponding points was implemented using the Insight Toolkit (ITK). All registrations were performed in the software package *elastix* (see http://elastix.isi.uu.nl). All programs were run on a Windows computer with an Intel Dual Core 2.40 GHz CPU and 64.0 GB memory.

### Data

The 4D cardiac data was acquired by a dual-source CT scanner (Siemens Somatom Definition, Germany). Fifteen patients were scanned with 10% R–R interval phases. They have confirmed pathologies including myocardium infarction, aortic valve stenosis, dilated cardiomyopathy, atrial fibrillation, and tricuspid regurgitation. The image dimensions were $512 \times 512 \times 131 \sim 265$ voxels of size $0.348 \times 0.348 \times 0.5$ mm. It is common to see that these cases displayed a wide diversity of heart shapes. To avoid the bias of using an atlas with similar heart shape, we constructed an atlas at each time phase from ten patients with all pathologies of above. The images from the remaining five patients are considered as unsegmented images.

The manual segmentations of all these data were performed as the gold standard. They were done by either a clinician or a researcher with knowledge of heart anatomy using an open-source tool ITK-SNAP (see http://www.itksnap.org). During this operation, right atrium (RA) and right ventricle (RV) were delineated firstly. Then left ventricle myocardium (LVM) and the blood cavities of left atrium (LA), left ventricle (LV) were separately segmented. The region of aorta (AO) was generated in the end (examples of manual segmentation can be found in Fig. 6). There are ten atlas intensity images and their label images, while the unsegmented images consist of fifty volume data. In total fifty registrations were performed for our proposed algorithm.

### Choice of parameters

In extraction procedure of the point pairs, we have determined some parameters empirically for the good result. For multi-scale Harris corner detector, the integration scale was set to $\upsigma_{\text{I}} = 1.5 \times 2^{i} (i = 0, \ldots , 4)$ and $\upsigma_{\text{D}}   = 0.7\sigma_{I}$. The threshold parameters were set to ${\text{T}}_{1} = {\text{T}}_{2} = 0.1$ with $\upalpha = 0.04$. For local extrema detector in the DoG space, the scale factor was set to 2, the scale for Gaussian blur was set to 1.5, and $T_{3} = 0.0075$. We selected $T_{4} = 0.9$ for feature matching using *n*-SIFT descriptor.

An affine initial registration was performed before nonrigid registration using the B-splines FFD. To avoid the local extrema, we employed a multi-resolution scheme with four levels. Gaussian smoothing instead of downsampling was applied to the moving images, with $\upsigma = 8.0, 4.0, 2.0,$ and 1.0 voxels for *x*, *y*, and *z* directions. As for the B-spline control points, the grid spacing of 80, 40, 20, and 10 mm in all directions was applied to four resolution levels respectively. A value of $\upomega = 0.01$ can provide a good balance between the two terms of the cost function. During the parameter optimization, $A = 50$, $\tau = 0.602$, $a = 2000$ were set, as well as 1000 iterations were used.

### Evaluation method

In order to validate the new method, we compared this hybrid registration (called MI + CP) with original MI and CC. Automatic segmentations of six substructures were generated by transforming atlas label images, with registration results. Two types of measures were used to evaluate the accuracy of segmentation result. The Dice similarity coefficient (DSC) [28] was calculated between the transformed segmentation (*V*_*seg*_) and the gold standard (*V*_*gd*_)

9 $$ DSC = \frac{{2\left| {V_{seg}  \mathop \cap \nolimits  V_{gd} } \right|}}{{\left| {V_{seg} } \right| + \left| {V_{gd} } \right|}} $$

where $\left| \cdot \right|$ denotes the number of voxels within the segmentation. ${\text{DSC}} = 0$ indicates no overlap while ${\text{DSC}} = 1$ indicates perfect agreement.

To compare segmentation accuracy of the two approaches, two-sided Wilcoxon tests [29] were carried on the corresponding DSC values. A value of $p < 0.05$ was regarded as a statistically significant difference. The surface distance measures compute the shortest distance between each surface point from the transformed segmentation ($x_{seg}  (i)$) and the surface of gold standard segmentation (*x*_*gd*_)

10 $$ d_{i} = min\left( {\left| {x_{seg} \left( i \right) - x_{gd} } \right|} \right) $$

where $\left| { \cdot } \right|$ is absolute value operator. It can provide insight into the spatial distribution of the registration errors.

## Results

**Fig. 5:**
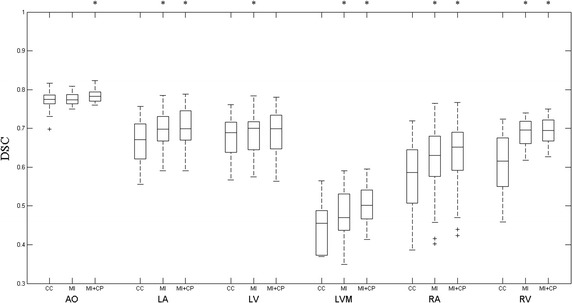
The boxplot of DSC results using the three methods for six substructures. A *star* indicates a statistical significant difference of the median overlap compared to the previous column

Figure 5 plots the Box-and-Whisker diagram of all registrations. The result of CC is worst in the three methods. Compared to MI, the median overlap of MI + CP increases significantly from 0.7734 to 0.7826 ($p = 3.0 \times 10^{ - 4}$) at the AO, from 0.6978 to 0.6991 ($p = 1.6 \times 10^{ - 2}$) at the LA, from 0.4703 to 0.5015 ($p = 5.0 \times 10^{ - 4}$) at the LVM, from 0.6307 to 0.6519 ($p = 6.0 \times 10^{ - 4}$) at the RA, and from 0.6947 to 0.6962 ($p = 3.3 \times 10^{ - 3}$) at the RV. At the LV, no much difference is seen between the two methods (from 0.6999 to 0.7000, $p = 5.0 \times 10^{ - 1}$).

**Fig. 6:**
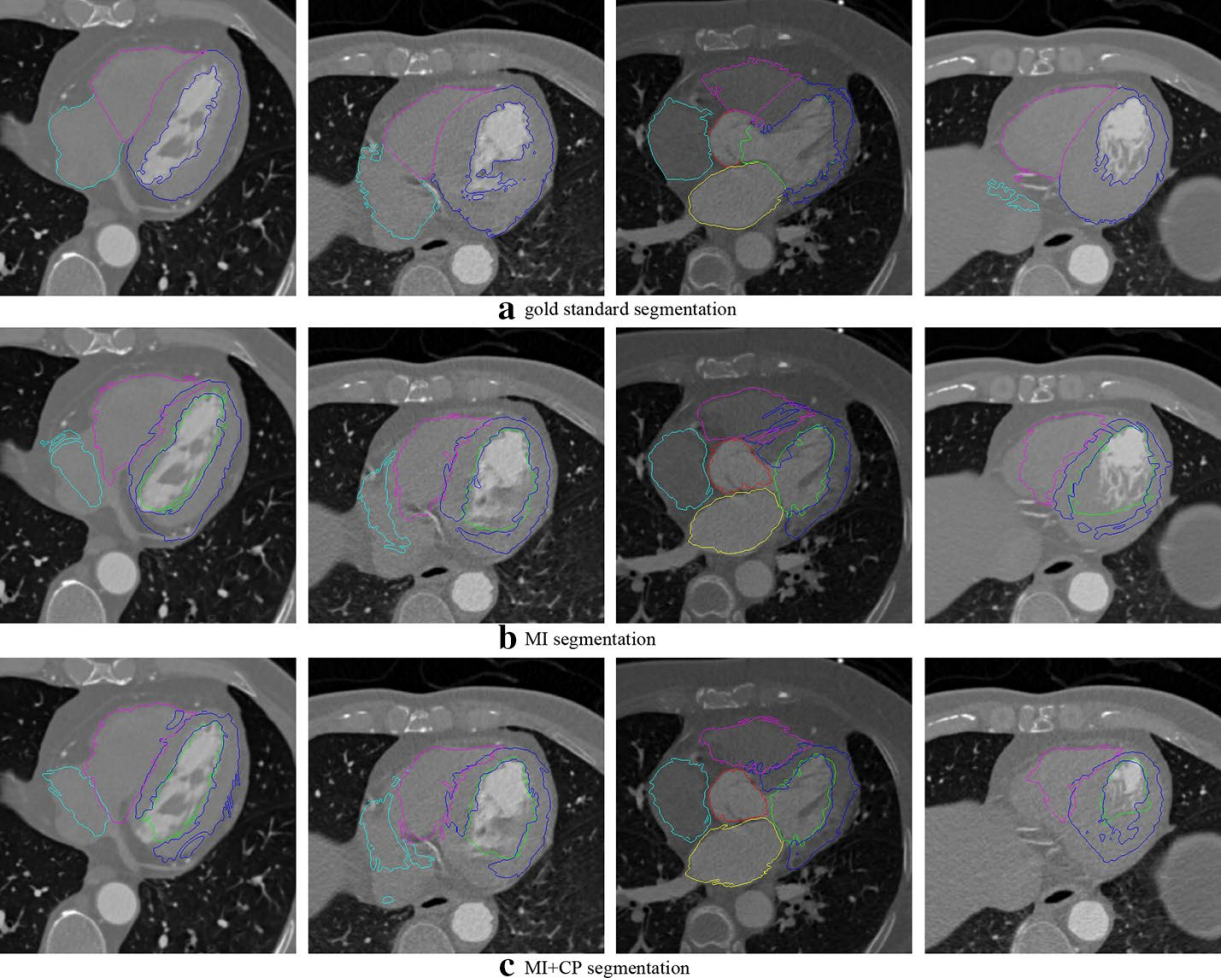
The exhibition of the segmentation results of four cases by the gold standard segmentation (*top row*), MI method (*middle row*), and MI + CP method (*bottom row*)

Table 1 lists the mean and standard deviation of the 50 cases. It agrees with the results in Fig. 5. The segmentation of the Aorta was better than other substructures. The worst result was at the left ventricle myocardium. Figure 6 displays the segmentation results of four cases by the gold standard segmentation and the two methods. The result of using MI + CP method is clearly closer to the gold standard than that of using MI method. It means that less manual correction is needed using MI + CP, if the technique was to be used in the clinic.

**Table 1:** The mean and standard deviation of DSC results using the three methods for six substructures

Structures	Methods	DSC (mean ± SD)	Cohen’s *d*
AO	CC	0.7716 ± 0.0006	–
	MI	0.7759 ± 0.0150	0.410
	MI ± CP	0.7826 ± 0.0150	0.452
LA	CC	0.6643 ± 0.0035	–
	MI	0.6980 ± 0.0460	1.044
	MI ± CP	0.7027 ± 0.0490	0.100
LV	CC	0.6769 ± 0.0030	–
	MI	0.6863 ± 0.0520	0.258
	MI ± CP	0.6901 ± 0.0520	0.073
LVM	CC	0.3731 ± 0.0346	–
	MI	0.4768 ± 0.0630	2.061
	MI ± CP	0.5076 ± 0.0490	0.551
RA	CC	0.5657 ± 0.0099	–
	MI	0.6180 ± 0.0890	0.834
	MI ± CP	0.6332 ± 0.0820	0.179
RV	CC	0.6076 ± 0.0052	–
	MI	0.6904 ± 0.0330	3.566
	MI ± CP	0.6935 ± 0.0320	0.096

Cohen’s *d* value from the current method compared to the method in previous row

**Fig. 7:**
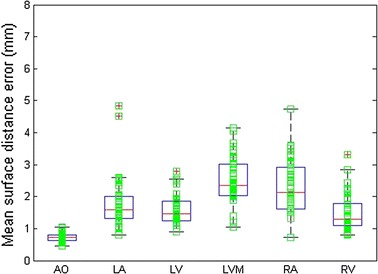
The boxplot of the mean surface distance using MI + CP for each substructure

**Fig. 8:**
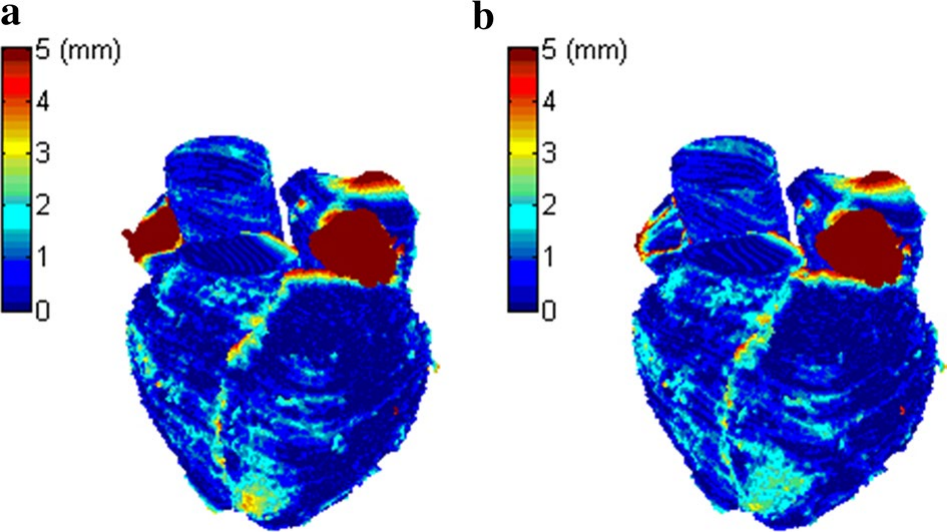
The error map of surface distance for the whole heart segmentation by the two methods. **a** Using MI. **b** Using MI + CP

The boxplot of the mean surface distance using the proposed method for six substructures is shown in Fig. 7. Table 2 also lists the mean and standard deviation of surface distance error for each substructure. It is obvious that most of them are below 2.0 mm, except left ventricle myocardium and right atrium. This was because there was no clear boundary between left ventricle myocardium and right ventricle, right atrium and right ventricle in some cases. Figure 8 shows the color map of surface distance for the whole heart segmentation by the two methods. For right atrium and left ventricle myocardium it can be seen that the segmentation error of MI + CP is less than that of MI.

**Table 2:** The mean and standard deviation of surface distance measure using MI + CP for six substructures

Structures	Surface error (mm)
AO	0.73 ± 0.13
LA	1.78 ± 0.78
LV	1.60 ± 0.13
LVM	2.47 ± 0.72
RA	2.30 ± 0.79
RV	1.50 ± 0.62

## Discussion

In the experiments, six substructures were extracted to support quantitative evaluation of cardiac functions. The proposed MI + CP approach achieves more accurate segmentation result as the multi-scale Harris corner and extrema detector are adopted. In Fig. 4 it can be observed that these corresponding points are nonuniformly scattered on some substructures of cardiac images. This will result in the small Cohen’s *d* values of some substructures, such as left ventricle and right ventricle. One possible solution is to add some corresponding points on the edge of the specific substructures, since it can reduce local minima more directly. Another possibility is to employ some physical transformation models [30] to accommodate the large cardiac variations between two cases, which will at the same time make the proposed method more effectively.

There are two limitations of this work. Firstly, we cannot investigate the sensitivity of this framework for different atlases. It is a challenge to construct the comprehensive atlases on large and highly variable image datasets. In [6], Hoogendoorn et al. utilized spatio-temporal statistical model of the human heart based on 4D multislice CT to synthesize the high resolution atlas. This method isn’t suitable for the modeling of all cardiac substructures although it may reduce the segmentation errors. Another way is to employ a parameter-free approach that directly produces a vector field, such as the diffeomorphic demons [31]. Secondly, further improvement in computational accuracy is still required. At the left ventricle myocardium, the DSC result using the proposed framework is only 0.5076 ± 0.0490. It indicates that depending on only this propagation framework is insufficient to handle this substructure. Perhaps incorporating the boundary-based segmentation technique [32] into this process will improve this limitation.

## Conclusions

We have introduced a novel registration algorithm for the implementation of the heart segmentation-propagation framework. Our aim is to improve the segmentation accuracy of DSCT images under the condition of the large variations and boundary ambiguity. An extension of *n*-SIFT method was developed to generate the corresponding points from atlas and unsegmented image. Nonrigid registration was achieved by mutual information with corresponding points constraint based on the free-form deformation. We have tested the performance using 4D cardiac images of fifteen patients. It was shown that the median overlap of our method improves significantly on most of anatomical substructures except left ventricle, in comparison to original mutual information. The reason should be that there are no enough corresponding points to support this substructure. In fact, the segmentation of the left ventricle is more challenging than the right ventricle and other parts because large displacements frequently occur between adjacent images or the papillary muscles fuse with the wall [33]. The segmentation errors had been significantly reduced by the proposed algorithm, in particular left ventricle myocardium and right atrium. The proposed segmentation framework achieved a mean surface distance of 1.73 mm for the whole heart between the propagated segmentation and the gold standard segmentation.

In future work, the diffeomorphic demons model could be used for atlas construction. It could be valuable to further investigate the effect of different atlases on the segmentation-propagation framework. Additionally, it is also important to develop the approach that enables us to propagate the atlas from a cardiac phase to another cardiac phase.

## Abbreviations

CVDs: cardiovascular diseases
MRI: magnetic resonance imaging
CT: computed tomography
MSCT: multislice CT
DSCT: dual-source CT
PCA: principal component analysis
*n*-SIFT: *n*-dimensional scale invariant feature transform
FFD: free-form deformation
α-MI: α-mutual information
DoG: difference of gaussian
ITK: Insight Toolkit
AO: aorta
LA: left atrium
LV: left ventricle
LVM: left ventricle myocardium
RA: right atrium
RV: right ventricle
DSC: dice similarity coefficient
